# Altered Expression of a Unique Set of Genes Reveals Complex Etiology of Schizophrenia

**DOI:** 10.3389/fpsyt.2019.00906

**Published:** 2019-12-12

**Authors:** Ashutosh Kumar, Vikas Pareek, Himanshu N. Singh, Muneeb A. Faiq, Ravi K. Narayan, Khursheed Raza, Pavan Kumar

**Affiliations:** ^1^Department of Anatomy, All India Institute of Medical Sciences (AIIMS), Patna, India; ^2^Department of Anatomy, All India Institute of Medical Sciences (AIIMS), New Delhi, India; ^3^Etiologically Elusive Disorders Research Network (EEDRN), New Delhi, India; ^4^Computational Neuroscience and Neuroimaging Division, National Brain Research Centre (NBRC), Manesar, India; ^5^TAGC—Theories and Approaches of Genomic Complexity, Aix Marseille University, Inserm U1090, Marseille, France; ^6^Neuroimaging and Visual Science Laboratory, New York University (NYU) Langone Health Centre, NYU Robert I. Grossman School of Medicine, New York, NY, United States; ^7^Developmental Neurogenetics Lab, Department of Pediatrics, Medical University of South Carolina, Charleston, SC, United States

**Keywords:** genome-wide expression study, genetic signature, hippocampus, associative striatum, prefrontal cortex

## Abstract

**Background:** The etiology of schizophrenia is extensively debated, and multiple factors have been contended to be involved. A panoramic view of the contributing factors in a genome-wide study can be an effective strategy to provide a comprehensive understanding of its causality.

**Materials and Methods:** GSE53987 dataset downloaded from GEO-database, which comprised mRNA expression data of post-mortem brain tissue across three regions from control (C) and age-matched subjects (T) of schizophrenia (N = Hippocampus [HIP]: C-15, T-18, Prefrontal cortex [PFC]: C-15, T-19, Associative striatum [STR]: C-18, T-18). Bio-conductor—affy—package used to compute mRNA expression, and further t-test applied to investigate differential gene expression. The analysis of the derived genes performed using the PANTHER Classification System and NCBI database. Further, a protein interactome analysis of the derived gene set was performed using STRING v10 database (https://string-db.org/)

**Results:** A set of 40 genes showed significantly altered (p < 0.01) expression across all three brain regions. The analyses unraveled genes implicated in biological processes and events, and molecular pathways relating basic neuronal functions.

**Conclusions:** The aberrant expression of genes maintaining basic cell machinery explains compromised neuronal processing in SCZ.

## Introduction

The etiology of schizophrenia (SCZ) is extensively debated (1, 2). Uncertainty of the etiology has greatly impeded the treatment of the disease, and neither of the therapeutic approaches (1) is proving much helpful in halting its progression.

Many candidate genes have been reported (3) for SCZ but none of them got validated in population-based studies for persistent association (4, 5). A disease signature derived from genome-wide expression patterns in affected brain regions was highly desirable that would help to reach the diagnosis and developing optimal therapeutic approaches for SCZ.

SCZ gives a life time risk of ∼1% and shows high heritability (∼69–81%) (6–8). The SCZ heritability is derived from CNVs, SNPs, *de novo* mutations, and structural modifications at gene promoter regions without involving gene sequences as have been revealed in the genome-wide studies (9). Expression derangement of the genes also evidenced to arise of the gene- environment interactions during fetal development and in the lifetime of the individuals (10, 11).

SCZ has been noted to cause significant architectural changes in many brain regions, the hippocampus, prefrontal cortex, and basal nuclei (more specifically associative or dorsal striatum) have been chief among them (12–14). Neuronal gene expressions in the affected brain regions are known to alter in SCZ (15, 16). A comprehensive study of the gene expression dynamics in SCZ patients, which is shared by all three brain regions (hippocampus, prefrontal cortex, and associative striatum) may plausibly give a glimpse of the disease etiology.

How the neural architectural changes are instructed by the changes in the neural genes has also been shown by some recent studies. Piskorowski et al. (17) have shown in the mouse model that deletion of 22q11 locus may involve the genes making synaptic proteins and that may produce SCZ like symptoms (17). Fromer and colleagues (18) have identified over 100 of genetic loci harboring SCZ associated variants which together involve scores of genes, and altering the expression or knock down of some of such genes in animal or human stem cell models has shown to compromise neural functions effectively (18). Plausibly, the dysregulation of the neuronal genes, especially which are involved in maintaining basic cell architecture and machinery, may compromise the information processing in neurons in affected brain regions in SCZ (19, 20).

In this study, we hypothesized that the contributory factors involved in the etiogenesis of SCZ may get reflected in the altered expression of neuronal genes; hence an ontological analysis of these genes from the affected brain regions may unravel the components of the complex etiology of SCZ.

## Materials and Methods

### Data Resources

The mRNA expression data were retrieved from the GEO (Genome Expression Omnibus, GSE53987) (http://www.ncbi.nlm.nih.gov/geo/), a public repository for high-throughput microarray. The RNA was originally isolated from post-mortem brain tissue across three specific regions (hippocampus [HIP], prefrontal cortex [PFC]: Brodmann Area 46 [dorsolateral part of PFC], and Associative [or dorsal] striatum [STR]) of control (N = 18 [HIP], 19 [PFC], 18[STR]) and age-matched subjects with schizophrenia (N = 15 [HIP], 15 [PFC], 18 [STR]). For the original data, designated tissue samples were acquired from the curated collection of brain samples from the University of Pittsburgh which was permitted by the institute ethics committee (21, 22). Equal numbers of male and female (except for odd number samples) diagnosed SCZ cases and controls of adult age were chosen for this purpose (**Table S1**). The controls were matched for the age and sex with cases, and were free of any neurological or psychiatric illness during their life course. Tissue was collected from same hemisphere of the brain using same anatomical landmarks in all individuals. The post-mortem interval (PMI) and pH of the brain tissue, storage timing (at −80 degree Celsius), and RNA integrity number (RIN) (to confirm quality of processed RNA) for the test and controls were maintained to the set standard as were declared in the published records related to the original data source (21, 22). Also, history of taking tobacco products, centrally acting drugs, and any other medications and manner of death for the tests as well control samples were noted from the records (**Table S1**) (21, 22).

### Data Retrieval and Analysis

The RNA was isolated from HIP, PFC (Brodmann Area 46), and associative STR and hybridized to U133_Plus2 Affymetrix chips for m-RNA expression study (21, 22). Expression analysis of mRNA was done by using "affy" package (http://www.bioconductor.org/packages/release/bioc/html/affy.html), which was deposited at Bioconductor and developed in R statistical software program and scripting language. It used three steps to calculate the expression intensities: (i) background correction; (ii) normalization (data were normalized by RMA, subjected to pair wise comparison followed by Benjamini and Hochberg False Discovery rate correction [FDR]), and (iii) expression calculation. After calculation of mRNA expression intensity, a simple unpaired two tailed t-test (significance set at p ≤ 0.01) was applied to the data to filter out the set of genes expressed significantly in all three brain regions.

To categorize the derived significantly altered genes on the basis of their involvement in molecular functions, molecular pathways, and biological events, PANTHER (Protein ANalysis THrough Evolutionary Relationships) Classification System (http://www.pantherdb.org/) and NCBI gene database (http://www.ncbi.nlm.nih.gov/gene/) were exploited. To construct a protein interactome network of the derived gene set STRING v10 database was used (https://string-db.org/). The pathway enrichment analysis results were extracted using Reactome Pathways of the STRING utility.

## Results

A set of 40 genes (protein coding-38; RNA-gene-2) was identified showing statistically significant (p ≤ 0.01) altered mRNA expression in schizophrenic patients in the all three brain regions studied (**Table 1**). Interestingly, it was observed that most of the genes were down-regulated in all three brain regions (32/40). Also, the same genes in all three brain regions have shown the similar direction of expression changes.

**Table 1:** Genome wide m-RNA expression (statistical significance set at p ≤ 0.01) in three brain regions of schizophrenic patients and healthy controls (data represented as mean).

		Hippocampus				Prefrontal cortex				Associative striatum		
Gene symbol	Control	SCZ	Fold change	p-value	Control	SCZ	Fold change	p-value	Control	SCZ	Fold change	p-value
	(n = 15)	(n = 18)	(Control/SCZ)		(n=15)	**(n = 19)**	(Control/SCZ)		(n = 18)	(n = 18)	(Control/SCZ)	
MSANTD3	8.33	8.03	0.96	0.001	8.71	8.71	0.98	0.003	8.15	8.03	0.99	0.006
CXADR	5.52	5	0.9	0.001	5.46	5.46	0.93	0.003	5.83	5.44	0.93	0.004
ZNF385B	6.27	5.47	0.87	<0.001	8.3	8.3	0.97	0.005	8.44	7.61	0.9	0.008
SAFB2	7.12	7.46	1.05	0.005	6.88	6.88	1.04	0.002	7.49	7.74	1.03	0.006
FBXO9 1559094_at	9.49	8.71	0.91	<0.001	10.19	10.19	0.95	0.002	8.78	8.28	0.94	0.002
FBXO9 1559096_x_at	10.68	10.07	0.94	<0.001	11.25	11.25	0.97	0.004	10.27	9.95	0.97	0.003
MCL1	9.74	10.23	1.05	0.002	9.76	9.76	1.03	0.002	9.83	10.25	1.04	0.002
PITPNA	10.74	10.39	0.96	<0.001	11.18	11.18	0.99	0.006	10.31	10.16	0.99	0.008
PSMC3	9.64	9.17	0.95	<0.001	9.92	9.92	0.98	0.008	10.15	9.8	0.97	0.008
IFITM2	9.77	10.46	1.07	0.001	9.29	9.29	1.06	0.003	10.04	10.47	1.04	0.008
ICMT	8.09	7.93	0.98	0.008	8.22	8.22	0.97	0.001	8.83	8.43	0.95	<0.001
UQCRC1	10.7	10.29	0.96	<0.001	11.06	11.06	0.99	0.003	10.69	10.46	0.98	0.008
ASNA1	9.3	8.99	0.96	<0.001	9.42	9.42	0.99	0.006	9.19	8.99	0.98	0.008
TIAL1	7.97	7.7	0.96	<0.001	7.94	7.94	0.98	0.003	7.89	7.67	0.97	0.003
GTF2H1	7.59	7.02	0.92	<0.001	8.04	8.04	0.97	0.002	7.38	6.96	0.94	0.003
ANPEP	9.22	10.26	1.11	< 0.001	8.81	8.81	1.09	0.004	9.36	10.11	1.08	0.005
SOX9	9.39	9.7	1.03	0.008	9.07	9.07	1.05	0.001	9.41	9.7	1.03	0.002
CHSY1	8.18	8.64	1.05	0.001	8.29	8.29	1.04	0.002	8.2	8.49	1.03	0.007
BCL6	9.78	10.48	1.07	<0.001	10.18	10.18	1.03	0.002	9.52	10	1.05	0.009
PDCD6	10.11	9.65	0.95	<0.001	10.29	10.29	0.98	0.004	10.01	9.73	0.97	0.005
ERCC1	9.13	8.84	0.97	<0.001	9.09	9.09	0.98	0.008	8.85	8.65	0.98	<0.001
MYO5A	8.7	8.3	0.95	0.003	9.24	9.24	0.96	0.004	7.69	7.35	0.95	0.002
KCNK1	10.73	10.4	0.97	0.003	10.9	10.9	0.98	0.003	10.08	9.78	0.97	0.008
GNAO1	10.58	10.23	0.96	0.006	10.61	10.61	0.97	0.001	10.37	10.03	0.97	0.005
HAPLN1	5.84	5.14	0.88	0.002	5.88	5.88	0.94	0.002	4.77	4.49	0.94	0.007
PPM1E	9.12.	8.26	0.9	0.007	7.62	7.62	0.96	0.008	6.37	6.08	0.95	0.001
KAT5.	8.46	8.29	0.97	0.001	8.7	8.7	0.99	0.008	8.68	8.56	0.99	0.008
MAPK9	8.13	7.5	0.92	<0.001	8.74	8.74	0.96	0.001	8.03	7.63	0.95	0.003
SPAG7	9.9	9.65	0.97	<0.001	9.92	9.92	0.99	0.001	9.52	9.37	0.98	0.001
ANAPC5	11.02	10.84	0.98	<0.001	10.95	10.95	0.99	0.001	11.02	10.8	0.98	0.009
ATP5D	10.71	10.4	0.97	<0.001	10.76	10.76	0.98	0.005	10.66	10.46	0.98	0.008
UBE4B	7.76	7.5	0.96	0.006	8.03	8.03	0.98	0.01	7.71	7.45	0.97	0.004
SCRN3	8.12	7.65	0.94	0.001	8.18	8.18	0.98	0.008	7.64	7.27	0.95	0.002
SMIM7	9.13	8.77	0.96	0.001	9.35	9.35	0.98	0.005	9.38	9.05	0.96	0.002
PDK4	8.24	9.15	1.11	0.001	7.98	7.98	1.11	<0.001	8.6	9.27	1.07	0.003
RBM18	9.31	8.99	0.96	0.001	9.6	9.6	0.99	0.006	9.18	8.96	0.98	0.003
SAMD5	6.76	6.38	0.94	0.006	6.85	6.85	0.96	0.001	7.47	6.85	0.92	<0.001
CADPS	6.43	6.2	0.96	0.004	6.4	6.4	0.96	0.005	6.1	5.89	0.97	0.004
LOC100506538///NDUFAF6	8.13	7.57	0.94	0.001	8.5	8.5	0.97	0.004	8.11	7.69	0.95	0.008
LOC100507534	6.38	5.28	0.83	0.002	6.29	6.29	0.88	< 0.001	4.42	4.19	0.95	0.007

**Figure 1:**
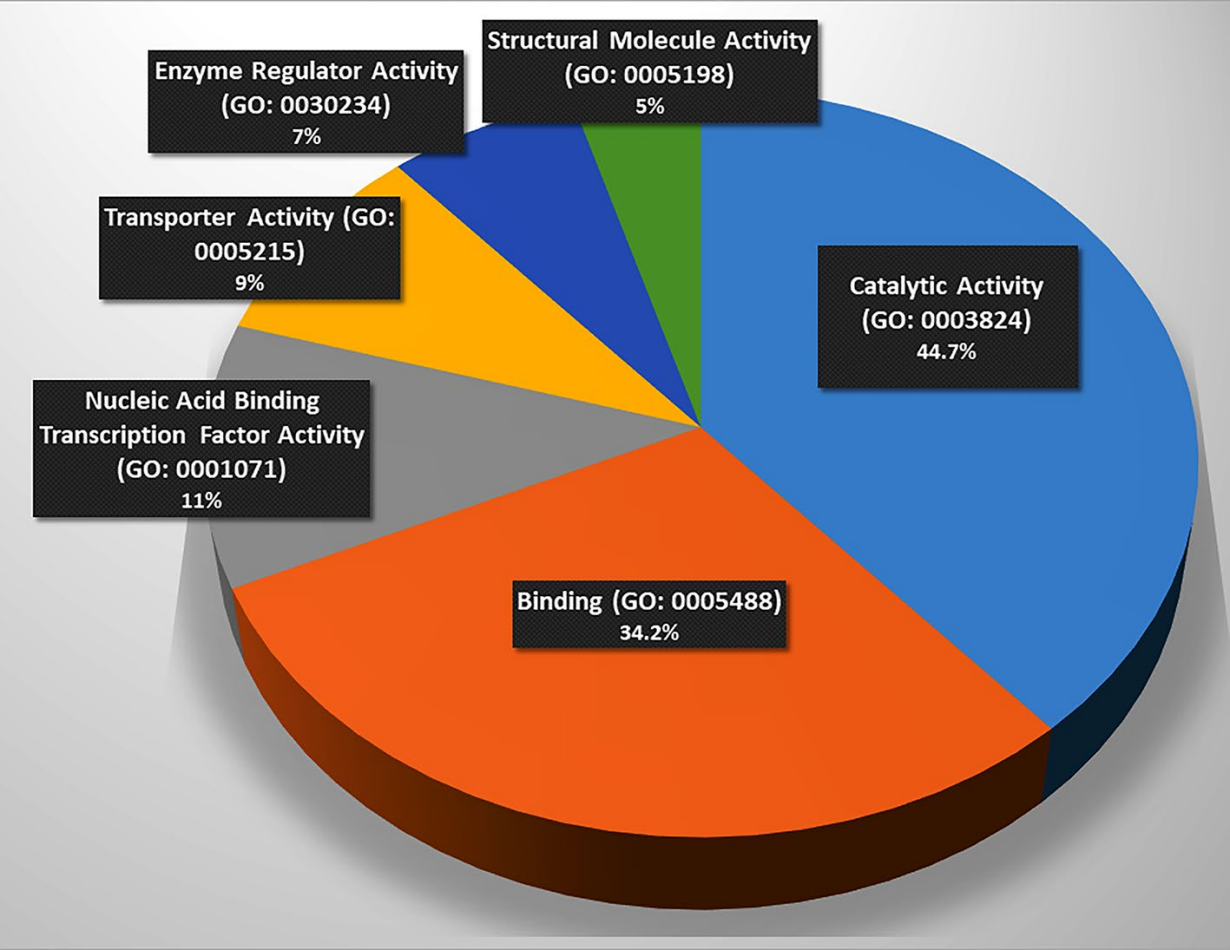
Involvement of the gene set in molecular functions. (Catalysis (n = 17, p = 44.7%), binding (n = 13, p = 34.2%), and nucleic acid binding transcription factor activity (n = 5, p = 13.2%), transporter activity (n = 4, p = 10.50%), enzyme regulation (n = 3, p = 7.90%), and structural molecule activity (n = 2, p = 5.3%), n = number of genes, p = percentage. Source: Panther Classification System).

These genes were classified into six categories on the basis of their molecular functions (**Figure 1**). Further, the genes were classified into fourteen categories on the basis of involvement in biological processes and events (**Table 2**). However, some genes belong to more than one category. The protein interactome network analysis of the gene-set revealed weak to strong interaction between only limited genes (**Figure S1**). Two cluster hubs were identified where more than two genes were observed to be interacting with each other. The salient genes which showed cross-talk in the interactome were *ATP5D*, *UQCRC1* and *ANAPC5*, *PSMC3* which are mainly involved in the basic cellular functions like energy production mechanisms and cellular growth, cell cycle regulation, and enzyme activity (**Table 2**).

**Table 2:** Involvement of the gene set in biological processes and events.

**a**	**Ubiquitination**	FBXO9, ANAPC5, UBE4, SMIM7
**b**	**Enzyme activity**	PPM1E, MAPK9, ATP5D, ICMT, PDK4, PSMC3
**c**	**Energy production mechanisms**	UQCRC1, ASNA1, ATP5D, PDK4
**d**	**Cell growth**	MAPK9, ANAPC5, CHSY1
**e**	**Programmed cell death**	IFITM2^a^, TIAL1^a^, PDCD6^a^, MCL-1^b^
**f**	**Cytoplasmic vesicular transport and exocytosis**	MYO5A, PITPNA, ASNA1, CADPS, SCRN3
**g**	**Dynamic regulation of cytoskeleton**	MYO5A
**h**	**Ion channel homeostasis**	KCNK1, PDCD6
**i**	**Lipid binding and synthesis**	PITPNA, CAPDS
**j**	**DNA repair**	ERCC1, KAT5
**k**	**m-RNA transcription**	MSANTD3, GTF2H1, GNAO1, MAPK9
**l**	**Post transcriptional gene modifications**	KAT5
**m**	**Protein translation**	TIAL1
**n**	**Cell cycle regulation**	ANAPC5, MAPK9

NCBI gene database (http://www.ncbi.nlm.nih.gov/gene/). a = Pro-apoptotic, b = Anti-apoptotic.

**Figure 2:**
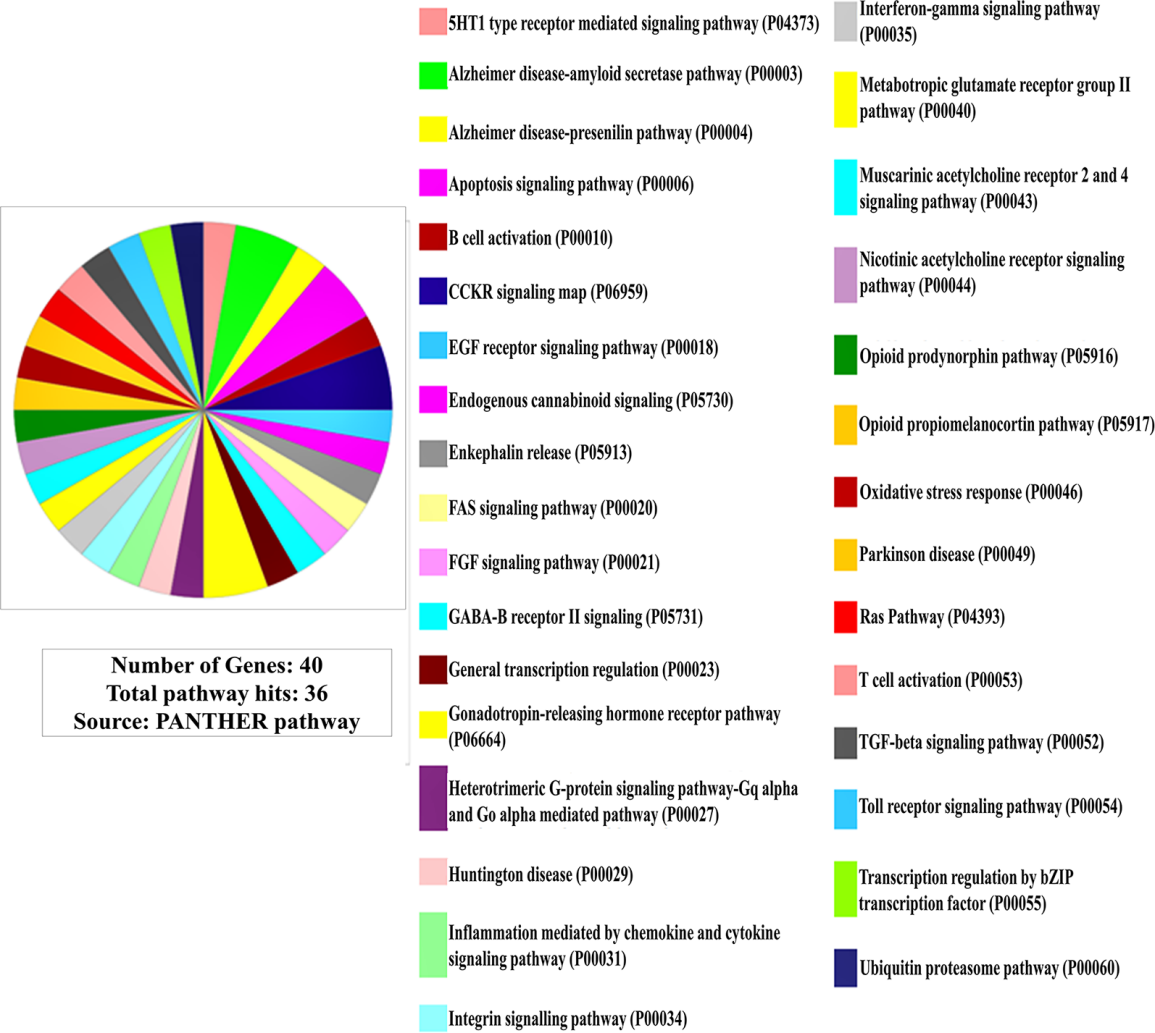
Involvement of the gene set in molecular pathways (Out of 40 genes only 38 are protein coding for which pathway involvements are known and has been presented here).

Furthermore, in pathway linkage analysis, the gene set was found to link with 36 molecular pathways (**Figure 2**) that broadly could be placed in seven categories based on their commonality (**Table S2**).

## Discussion

The structural and functional brain abnormalities have been repeatedly reported in patients with SCZ (23). The brain regions chosen for this study (hippocampus, prefrontal cortex, and striatum) are known to be predominantly affected (12–14) and their dysfunctions (contributing to characteristic symptoms as altered cognition, loss of executive functions, and disorganized thought and behavior) are common in SCZ (24–26). Recent studies supported an altered gene expression in of these brain regions in SCZ (27, 28). A comprehensive study of the genome expression status in these brain regions was expected to unravel mysterious etiology of SCZ.

As the genes selected for the analysis were found significantly altered in all three studied brain regions, these expression changes may be reflecting the actual pathophysiological changes occurring in neuronal functions in SCZ. None of the genes revealed in this study had been reported earlier as a candidate gene for SCZ, hence the new set appealed for a fresh attention to understand the disease etiology.

### Involvement of the Gene Set in Molecular Functions

The functional analysis of the gene-set (**Figure 1**) elucidated the genes being involved in regulation of basic machinery and housekeeping functions of the neurons *viz.* receptor-ligand binding, catalysis, enzymatic regulation, nucleic acid binding transcription factor activity, structural molecule activity, and transport activities. It is well evident that dysregulation of these basic functions in neurons may manifest in compromised information processing in brain which has been a hallmark of the progressed SCZ (29). The molecular function analysis also showed hierarchy of the functions that may be compromised in SCZ (**Figure 1**) (the catalysis and receptor-ligand binding being most affected functions).

### Involvement of the Gene Set in Biological Processes and Events

The comprehensive influence of the dysregulation of these genes in the pathogenesis of SCZ gets further clarified in the analysis for the involvement in the biological processes and cellular events (**Table 2**). The implication of genes involved in ubiquitination (**Table 2-a**), enzyme activity (**Table 2-b**), and energy production mechanisms (**Table 2-c**) may point towards a failure of the basic functions in neurons; as ubiquitination is known to regulate the diverse spectrum of cellular functions (30) and the same should be true for the genes encoding enzymes, especially those necessary for mitochondrial functions (*ATP5D*, *PDK4*) (31), regulating specific signaling pathways (*MAPK9*) (32) and involved in phosphorylation (*ATP5D*, *PDK4*) or dephosphorylation (*PPM1E*) (25, 33). The dysregulation of genes involved in energy production (**Table 2-c**) supports prevailed view in the literature that energy production mechanisms get compromised in SCZ (34, 35).

Furthermore, down-regulation of genes which function as regulator of the cell growth mechanisms (**Table 2-d**) provides a possible explanation for reduced neuronal cell sizes, synaptic connection and brain volume in specific brain regions noted in schizophrenia (36) The significant upregulation of genes involved in the programmed death (**Table 2-e**) may indicate pro-apoptotic mechanisms prevailing in particular brain regions in schizophrenia which gets support from some earlier studies (37, 38). Though, the pro-apoptotic mechanisms may not be a generalized feature in SCZ, as we also noted contrary evidence that an anti-apoptotic gene *MCL-1* was found significantly upregulated in all three brain regions. [Contrastingly, *sMCL-1* regulates cell cycle negatively hence limiting the mitosis (39)].

Also, the significantly altered expression of the genes involved in cytoplasmic vesicular transport and exocytosis (**Table 2-f**), dynamic regulation of actin and tubulin cytoskeleton (**Table 2-g**), and ion channel homeostasis (**Table 2-h**), lipid-binding (PITPNA) and synthesis (CADPS) (**Table 2-i**) hint of compromised neuronal information processing in SCZ.

#### Indications From Interactome Analysis of Gene Set

The protein-protein interaction analysis of the gene set revealed cross-talk between *ATP5D* (**Table 2-b, c**), *UQCRC1* (**Table 2-c**), and *ANAPC5* (**Table 2-d, n**), *PSMC3* (**Table 2-b**) (**Figure S1**) which further strengthen the view that basic (neuronal) cell functionary of the selective brain regions could be the focus of the pathogenesis in SCZ. We detected two central hubs showing strong interaction between the genes involved in ubiquitination (*ANAPC5*), enzyme activity (*ATP5D*, *PSMC3*), energy production mechanism or mitochondrial functions (*ATP5D*), and cell cycle regulation (*ANAPC5*) (**Figure S1**). Existing literature suggests that a dysregulation of the noted biological processes and events could be at the core of SCZ pathogenesis (40–42).

### Involvement of the Gene Set in Molecular Pathways

In pathway linkage analysis (**Figure 2**, **Table S2**), the category involving largest number of molecular pathways has been that of neurotransmitters/modulators and neurohormones (**Table S2-a**) which fits with clinical manifestations of the disease and also gets support from existing theories that the etiology of SCZ majorly may be based on dysregulation of this category of molecules (43).

Various neurotransmitters-based hypotheses have been proposed for the etiology of SCZ (44) but none of them are primarily explaining causality of the diseases. The result of this study (**Figure 2**, **Table S2**) indicates that disease etiology is not implicating any single transmitter but many of them together (45).

The linkage of the immune cell/chemokine mediated pathways (**Table S2-b**) is strongly supported by literature (46, 47). An immunogenic basis of SCZ etiogenesis had also been brought forward (48) although counter to this hypothesis has also been placed which limits the role of immune function related genes as a solo or major factor in SCZ etiology (49).

In a recent study (21), Lanz et al. who performed pathway analysis of transcriptional profiles in post-mortem samples of HPC, PFC, and associative STR from SCZ patients (using the same dataset which we used in our study) found enrichment of the transcripts involved in inflammatory pathways. Enrichment of the inflammatory pathways was also reported by Scarr et al., who examined transcriptional profiles from PFC of post-mortem SCZ patients (50).

Also, the involvement of growth, differentiation, and survival of neurons in the specific brain regions (**Table S2-c**) (51, 52) (also discussed in subsection *Involvement of the Gene Set in Biological Processes and Events*) and pathways related to apoptosis (**Table S2-d**) (37, 38), and related to protein synthesis (**Table S2-e**) and degradation (**Table S2-f**) (53) has been well documented in the literature (also discussed in subsection *Involvement of the Gene Set in Biological Processes and Events*). The linkage of FGF signaling pathway (**Table S2-c**) under neuronal growth, differentiation, and survival to SCZ etiology has been corroborated by a freshly published study by Narla et al. (54) who regarded it as a central pathway commanding all other pathways in developing brain strengthening the view that SCZ has a neurodevelopmental etiology (54). In contrast, the linking of the pathways involved in the pathogenesis of major neurodegenerative diseases (**Table S2-g**) such as Alzheimer, Parkinson, and Huntington’s disease indicates neurodegenerative nature of SCZ.

### Non-Protein-Coding Genes: Unknown Functions

The neuronal functions associated with two non-coding genes (*LOC100507534*, *LOC100507534*) couldn’t be ascertained from the literature but it’s interesting to find significant alterations of these long non-coding RNAs in SCZ which have never been reported before. There are now strong indications that non-coding genes are implicated in SCZ pathology (55).

## Limitations of This Study and Further Research

The confounding factors such as the history of addiction (including alcohol intake) or substance abuse, and drug intake might have some impact on the transcriptional data. A separate analysis of the impacts of these confounding factors (especially that for addiction and substance abuse) might have given additional insights to this study. Additionally, a separate analysis of the impact of the sex of the subject on the resultant data could have provided important insights regarding sex-specific pathogenesis in SCZ. We couldn’t analyze these factors separately either due to the lack of related data or limitation of the sample sizes.

Though, we have included only those genes for further analysis which showed significant change of expression in all three studied brain regions, the fold changes for the genes are not very large to derive strong conclusions. Validation of the analyzed data with more than one gene expression analysis methods could have been necessary, and could have further augmented the value of this study. The genes which were significantly altered in only one or two and not in all three brain regions selected for the study have not been included in analysis to keep the study design robust, but they might carry some value in disease etiology. A neural circuit specific analysis of the changes in gene expressions targeted to the individual neurocognitive domains may further enhance the etiological clarity on SCZ.

Testing validity of the proposed gene set as a SCZ genetic signature is a remaining task which needs to be studied further. A rigorous search of the SCZ gene expression databases linked to the noted brain regions will make this clear. Also, looking for the similar gene expression changes in the blood cells and/or skin fibroblasts (though there is limited evidence for this in literature for now) (56, 57), will be greatly informative for assigning any prognostic (or diagnostic) value to the gene set.

## Conclusions

Molecular characterization of the gene set unraveled in this study gives a glimpse of the complex etiogenetic mechanisms involved in SCZ, an understanding of which may have useful implications in the therapeutic management of the disease. Most of the genes in the set participate in the maintenance of basic cell machinery which explains why their aberrant expression may cause compromised neuronal processing in SCZ.

## Data Availability Statement

The dataset used for this study can be found in the NCBI Genome Expression Omnibus, GEO accession: GSE53987 (https://www.ncbi.nlm.nih.gov/geo/query/acc.cgi?acc=GSE53987).

## Ethics Statement

Ethical review and approval was not required for the study on human participants in accordance with the local legislation and institutional requirements. Written informed consent for participation was not required for this study in accordance with the national legislation and the institutional requirements.

## Author Contributions

AK conceived and designed the study. HS, VP, and AK analyzed the data. AK wrote the first draft. AK, VP, HS, MF, RN, KR, and PK edited the first draft. AK, VP, and RN prepared final draft of the paper.

## Conflict of Interest

The authors declare that the research was conducted in the absence of any commercial or financial relationships that could be construed as a potential conflict of interest.

## Abbreviations

SCZ, Schizophrenia; HIP, Hippocampus; STR, Associative Striatum; PFC, Prefrontal Cortex.

## Supplementary Material

Table S1 Human subject demographics and sample metadata.

Table S2 Involvement of the gene set in molecular pathways.

Figure S1 Protein Interactome Network Analysis of the gene set. Certain of the genes shown in the figure, such as JADE-1 and ENSG00000251349 or MSANTD3-TMEFF1, PSMC6, PSMC2, ATP5F1, are linker genes generated by the database and are not part of the original gene set.

## References

[1] Rubeša G Gudelj L Kubinska N Etiology of schizophrenia and therapeutic options. Psychiatr Danub (2011) 23:308–15. 21963703

[2] Tandon R Keshavan MS Nasrallah HA Schizophrenia, “Just the Facts” What we know in 2008. 2. Epidemiology and etiology. Schizophr Res (2008) 102:1–18. 10.1016/j.schres.2008.04.011 18514488

[3] Gogos JA Gerber DJ Schizophrenia susceptibility genes: emergence of positional candidates and future directions. Trends Pharmacol Sci (2006) 27:226–33. 10.1016/j.tips.2006.02.005 16530856

[4] Crow TJ The emperors of the schizophrenia polygene have no clothes. Psychol Med (2008) 38:1681–5. 10.1017/S0033291708003395 18423075

[5] Farrell MS Werge T Sklar P Owen MJ Ophoff RA O’Donovan MC Evaluating historical candidate genes for schizophrenia. Mol Psychiatry (2015) 20(5):555. 10.1038/mp.2015.16 25754081 PMC4414705

[6] Lichtenstein P Yip BH Björk C Pawitan Y Cannon TD Sullivan PF Common genetic determinants of schizophrenia and bipolar disorder in Swedish families: a population-based study. Lancet (2009) 373(9659):234–9. 10.1016/S0140-6736(09)60072-6 PMC3879718 19150704

[7] Sullivan PF Kendler KS Neale MC Schizophrenia as a complex trait: evidence from a meta-analysis of twin studies. Arch Gen Psychiatry (2003) 60:1187–2. 10.1001/archpsyc.60.12.1187 14662550

[8] Wray NR Gottesman II Using summary data from the danish national registers to estimate heritabilities for schizophrenia, bipolar disorder, and major depressive disorder. Front Genet (2012) 3:118. 10.3389/fgene.2012.00118 22783273 PMC3387670

[9] Kavanagh DH Tansey KE O’Donovan MC Owen MJ Schizophrenia genetics: emerging themes for a complex disorder. Mol Psychiatry (2015) 20(1):72. 10.1038/mp.2014.148 25385368

[10] Caspi A Moffitt TE Gene–environment interactions in psychiatry: joining forces with neuroscience. Nat Rev Neurosci (2006) 7:583–90. 10.1038/nrn1925 16791147

[11] Champagne FA Early environments, glucocorticoid receptors, and behavioral epigenetics. Behav Neurosci (2013) 127:628–36. 10.1037/a0034186 24128352

[12] Kegeles LS Abi-Dargham A Frankle WG Gil R Cooper TB Slifstein M Increased synaptic dopamine function in associative regions of the striatum in schizophrenia. Arch Gen Psychiatry (2010) 67(3):231–9. 10.1016/S0920-9964(02)00294-3 20194823

[13] Manoach DS Prefrontal cortex dysfunction during working memory performance in schizophrenia: reconciling discrepant findings. Schizophr Res (2003) 60:285–8. 10.1176/appi.ajp.2010.09081187 12591590

[14] Tamminga CA Stan AD Wagner AD The hippocampal formation in schizophrenia. Am J Psychiatry (2010) 167:1178–93. 10.1038/tp.2016.173 20810471

[15] Darby MM Yolken RH Sabunciyan S Consistently altered expression of gene sets in postmortem brains of individuals with major psychiatric disorders. Transl Psychiatry (2016) 6:e890–0. 10.1038/tp.2016.173 PMC5048210 27622934

[16] Roussos P Katsel P Davis KL Siever LJ Haroutunian V A system-level transcriptomic analysis of schizophrenia using postmortem brain tissue samples. Arch Gen Psychiatry (2012) 69:1205. 10.1001/archgenpsychiatry.2012.704 22868662

[17] Piskorowski RA Nasrallah K Diamantopoulou A Mukai J Hassan SI Siegelbaum SA Age-dependent specific changes in area CA2 of the hippocampus and social memory deficit in a mouse model of the 22q11. 2 deletion syndrome. Neuron (2016) 89(1):163–76. 10.1016/j.neuron.2015.11.036 PMC4706988 26748091

[18] Fromer M Roussos P Sieberts SK Johnson JS Kavanagh DH Perumal TM Gene expression elucidates functional impact of polygenic risk for schizophrenia. Nat Neurosci (2016) 19(11):1442. 10.1001/archpsyc.59.7.631 27668389 PMC5083142

[19] Hemby SE Ginsberg SD Brunk B Arnold SE Trojanowski JQ Eberwine JH Gene expression profile for schizophrenia: discrete neuron transcription patterns in the entorhinal cortex. Arch Gen Psychiatry (2002) 59(7):631–40. 10.1038/mp.2009.18 12090816

[20] Maycox PR Kelly F Taylor A Bates S Reid J Logendra R Analysis of gene expression in two large schizophrenia cohorts identifies multiple changes associated with nerve terminal function. Mol Psychiatry (2009) 14(12):1083. 10.1038/s41398-019-0492-8 19255580

[21] Lanz TA Reinhart V Sheehan MJ Rizzo SJS Bove SE James LC Postmortem transcriptional profiling reveals widespread increase in inflammation in schizophrenia: a comparison of prefrontal cortex, striatum, and hippocampus among matched tetrads of controls with subjects diagnosed with schizophrenia, bipolar or major depressive disorder. Transl Psychiatry (2019) 9:151. 10.1038/s41398-019-0492-8 31123247 PMC6533277

[22] Lanz TA Joshi JJ Reinhart V Johnson K Grantham LEII Volfson D STEP levels are unchanged in pre-frontal cortex and associative striatum in post-mortem human brain samples from subjects with schizophrenia, bipolar disorder and major depressive disorder. PloS One (2015) 10(3):e0121744. 10.1016/j.schres.2003.12.002 25786133 PMC4364624

[23] Antonova E Sharma T Morris R Kumari V The relationship between brain structure and neurocognition in schizophrenia: a selective review. Schizophr Res (2004) 70(2-3):117–45. 10.3389/fpsyt.2013.00035 15329292

[24] Orellana G Slachevsky A Executive functioning in schizophrenia. Front Psychiatry (2013) 4:35. 10.3389/fpsyt.2013.00035 23805107 PMC3690455

[25] Simpson EH Kellendonk C Kandel E A possible role for the striatum in the pathogenesis of the cognitive symptoms of schizophrenia. Neuron (2010) 65:585–96. 10.1016/j.neuron.2010.02.014 PMC4929859 20223196

[26] Lieberman JA Girgis RR Brucato G Moore H Provenzano F Kegeles L Hippocampal dysfunction in the pathophysiology of schizophrenia: a selective review and hypothesis for early detection and intervention. Mol Psychiatry (2018) 23:1764–2. 10.1038/mp.2017.249 PMC6037569 29311665

[27] Faiz M Acarin L Castellano B Gonzalez B Proliferation dynamics of germinative zone cells in the intact and excitotoxically lesioned postnatal rat brain. BMC Neurosci (2005) 6:26. 10.1186/1471-2202-6-26 15826306 PMC1087489

[28] Mirnics K Middleton FA Marquez A Lewis DA Levitt P Molecular characterization of schizophrenia viewed by microarray analysis of gene expression in prefrontal cortex. Neuron (2000) 28:53–7. 10.1016/S0896-6273(00)00085-4 11086983

[29] Giersch A Poncelet PE Capa RL Martin B Duval CZ Curzietti M Disruption of information processing in schizophrenia: the time perspective. Schizophr Res: Cogn (2015) 2(2):78–3. 10.1146/annurev.biochem.67.1.425 PMC5609651 29114456

[30] Hershko A Ciechanover A The ubiquitin system. Annu Rev Biochem (1998) 67:425–9. 10.1186/s40591-016-0047-9 9759494

[31] Enriquez-Barreto L Morales M The PI3K signaling pathway as a pharmacological target in Autism related disorders and Schizophrenia. Mol Cell Ther (2016) 4:1. 10.1385/JMN:24:2:315 26877878 PMC4751644

[32] Bubber P Tang J Haroutunian V Xu H Davis KL Blass JP Mitochondrial enzymes in schizophrenia. J Mol Neurosci (2004) 24(2):315–21. 10.1523/JNEUROSCI.4650-03.2004 15456945

[33] Emamian ES Karayiorgou M Gogos JA Decreased phosphorylation of NMDA receptor type 1 at serine 897 in brains of patients with Schizophrenia. J Neurosci (2004) 24:1561–4. 10.1038/sj.mp.4001511 PMC6730463 14973229

[34] Prabakaran S Swatton JE Ryan MM Huffaker SJ Huang JJ Griffin JL Mitochondrial dysfunction in schizophrenia: evidence for compromised brain metabolism and oxidative stress. Mol Psychiatry (2004) 9(7):684. 10.1038/mp.2013.67 15098003

[35] Robicsek O Karry R Petit I Salman-Kesner N Müller FJ Klein E Abnormal neuronal differentiation and mitochondrial dysfunction in hair follicle-derived induced pluripotent stem cells of schizophrenia patients. Mol Psychiatry (2013) 18(10):1067. 10.1016/S0920-9964(96)00076-X 23732879

[36] Ward KE Friedman L Wise A Schulz SC Meta-analysis of brain and cranial size in schizophrenia. Schizophr Res (1996) 22:197–3. 10.1016/j.pnpbp.2005.03.010 9000317

[37] Jarskog LF Glantz LA Gilmore JH Lieberman JA Apoptotic mechanisms in the pathophysiology of schizophrenia. Prog Neuropsychopharmacol Biol Psychiatry (2005) 29(5):846–58. 10.1176/appi.ajp.161.1.109 15908096

[38] Jarskog LF Selinger ES Lieberman JA Gilmore JH Apoptotic proteins in the temporal cortex in schizophrenia: high Bax/Bcl-2 ratio without caspase-3 activation. Am J Psychiatry (2004) 161(1):109–15. 10.1074/jbc.M006626200 14702258

[39] Fujise K Zhang D Liu JL Yeh ET Regulation of apoptosis and cell cycle progression by MCL1 differential role of proliferating cell nuclear antigen. J Biol Chem (2000) 275(50):39458–65. 10.1038/s41598-019-38490-1 10978339

[40] Bousman CA Luza S Mancuso SG Kang D Opazo CM Mostaid MS Elevated biquitinated proteins in brain and blood of individuals with schizophrenia. Sci Rep (2019) 9(1):2307. 10.1186/1471-2164-15-S9-S6 30783160 PMC6381171

[41] Huang KC Yang KC Lin H Tsao TT Lee SA Transcriptome alterations of mitochondrial and coagulation function in schizophrenia by cortical sequencing analysis. BMC Genomics (2014) 15(9):S6. 10.1073/pnas.0903066106 PMC4290619 25522158

[42] Benes FM Lim B Subburaju S Site-specific regulation of cell cycle and DNA repair in post-mitotic GABA cells in schizophrenic versus bipolars. Proc Natl Acad Sci (2009) 106(28):11731–6. 10.1093/med/9780199378067.003.0010 PMC2703668 19564623

[43] Gill KM Grace AA The Role of Neurotransmitters in Schizophrenia. In: Schulz SC Green MF Nelson KJ, editors. Schizophrenia and Psychotic Spectrum Disorders. (Oxford, United Kingdom: Oxford University Press) (2016). p. 153–4. 10.1007/978-1-4613-4042-3_7

[44] Matthysse S Sugarman J Neurotransmitter Theories of Schizophrenia. In: Iversen LL Iversen SD Snyder SH, editors. Handbook of Psychopharmacology. (Basel, Switzerland: Springer) US (1978). p. 221–2. 10.1016/j.mehy.2012.01.035

[45] Bencherif M Stachowiak MK Kucinski AJ Lippiello PM Alpha7 nicotinic cholinergic neuromodulation may reconcile multiple neurotransmitter hypotheses of schizophrenia. Med Hypotheses (2012) 78(5):594–0. 10.2174/157339510791823673 22336089

[46] Müller N Schwarz MJ Immune system and Schizophrenia. Immunol Rev (2010) 6:213–20. 10.1186/1471-2202-12-13 PMC2971548 21057585

[47] Reale M Patruno A De Lutiis MA Pesce M Felaco M Di Giannantonio M Dysregulation of chemo-cytokine production in schizophrenic patients versus healthy controls. BMC Neurosci (2011) 12(1):13. 10.1038/s41537-017-0010-z 21266029 PMC3038147

[48] Malavia TA Chaparala S Wood J Chowdari K Prasad KM McClain L Generating testable hypotheses for schizophrenia and rheumatoid arthritis pathogenesis by integrating epidemiological, genomic, and protein interaction data. NPJ Schizophr (2017) 3(1):11. 10.1093/schbul/sbw059 28560257 PMC5441529

[49] Pouget JG Gonçalves VF Schizophrenia Working Group of the Psychiatric Genomics Consortium Spain SL Finucane HK Raychaudhuri S Genome-wide association studies suggest limited immune gene enrichment in schizophrenia compared to 5 autoimmune diseases. Schizophr Bull (2016) 42:1176–84. 10.1093/schbul/sbw059 PMC4988748 27242348

[50] Scarr E Udawela M Dean B Changed frontal pole gene expression suggest altered interplay between neurotransmitter, developmental, and inflammatory pathways in schizophrenia. NPJ Schizophr (2018) 4:4. 10.1038/s41537-018-0044-x 29463818 PMC5820249

[51] Lee AS De Jesús-Cortés H Kabir ZD Knobbe W Orr M Burgdorf C The neuropsychiatric disease-associated gene cacna1c mediates survival of young hippocampal neurons. Eneuro (2016) 3(2):0006-16. 10.1186/s12920-015-0098-9 PMC4819284 27066530

[52] Maschietto M Tahira AC Puga R Lima L Mariani D da Silveira Paulsen B Co-expression network of neural-differentiation genes shows specific pattern in schizophrenia. BMC Med Genomics (2015) 8(1):23. 10.1038/npp.2013.84 25981335 PMC4493810

[53] Rubio MD Wood K Haroutunian V Meador-Woodruff JH Dysfunction of the ubiquitin proteasome and ubiquitin-like systems in schizophrenia. Neuropsychopharmacology (2013) 38: (10):1910. 10.1016/j.schres.2016.12.012 23571678 PMC3746696

[54] Narla ST Lee YW Benson CA Sarder P Brennand KJ Stachowiak EK Common developmental genome deprogramming in schizophrenia—Role of Integrative Nuclear FGFR1 Signaling (INFS). Schizophr Res (2017) 185:17–2. 10.1016/j.celrep.2014.10.015 PMC5507209 28094170

[55] Roussos P Mitchell AC Voloudakis G Fullard JF Pothula VM Tsang J A role for noncoding variation in schizophrenia. Cell Rep (2014) 9(4):1417–29. 10.3389/fncel.2013.00095 PMC4255904 25453756

[56] Chana G Bousman CA Money TT Gibbons A Gillett P Dean B Biomarker investigations related to pathophysiological pathways in schizophrenia and psychosis. Front Cell Neurosci (2013) 7(0):95. 10.1371/journal.pone.0116686 23805071 PMC3693064

[57] Cattane N Minelli A Milanesi E Maj C Bignotti S Bortolomasi M Altered gene expression in schizophrenia: findings from transcriptional signatures in fibroblasts and blood. PloS One (2015) 10(2):e0116686. 10.1371/journal.pone.0116686 25658856 PMC4319917

