# Supplementary material for: Altered Expression of a Unique Set of Genes Reveals Complex Etiology of Schizophrenia
Source: Front Psychiatry. 2019 Dec 12;10:906. doi: 10.3389/fpsyt.2019.00906 (PMC6920214; doi:10.3389/fpsyt.2019.00906)
Supplement: Table S1 — Human subject demographics and sample metadata. [file Table_1.docx]

**Table S1. Human subject demographics and sample metadata.**

Values are mean ± standard deviation (n = 19 per group): RIN = RNA Integrity Number; Medication at time of death (Meds ATOD): C = centrally acting medications (Benzodiazepines, anticonvulsants, antidepressants, antipsychotics, lithium), O = other medications (for any ailment), U = unknown, Y=yes, N = none; MOD = Manner of Death (N = natural; A = accidental; S = suicide). Source: Lanz TA, et al. *PLoS One* 2015;10(3): e0121744. PMID: 25786133

|  | **Control** | **Schizophrenia** |
| --- | --- | --- |
| **Gender** | 10 M, 9 F | 10 M, 9 F |
| **Race** | 18 W, 1 B | 13 W, 6 B |
| **Age (years)** | 48.1 ± 10.6 | 45.1 ± 8.5 |
| **PMI (hours)** | 19.5 ± 5.1 | 20.1 ± 6.9 |
| **Brain pH** | 6.6 ± 0.2 | 6.4 ± 0.4 |
| **RIN** | 7.6 ± 0.67 | 7.0 ± 0.83 |
| **Tobacco ATOD** | 5 Y, 14 N | 12 Y, 7 N |
| **Meds ATOD** | 7 O, 12 N | 16 C, 1 O, 2 N |
