# Supplementary material for: Altered Expression of a Unique Set of Genes Reveals Complex Etiology of Schizophrenia
Source: Front Psychiatry. 2019 Dec 12;10:906. doi: 10.3389/fpsyt.2019.00906 (PMC6920214; doi:10.3389/fpsyt.2019.00906)
Supplement: Table S2 — Involvement of the gene set in molecular pathways. [file Table_2.docx]

**Table S2. Involvement of the gene set in molecular pathways**

| **Neurotransmitters/modulators/neuro-hormones** | **Immune cell/chemokine mediated** | **Growth, differentiation and cell survival** | **Apoptosis** | **Protein synthesis** | **Protein degradation** | **Pathogenesis of neurodegenerative diseases** |
| --- | --- | --- | --- | --- | --- | --- |
| **(a)** | **(b)** | **(c)** | **(d)** | **(e)** | **(f)** | **(g)** |
| (i)Metabotropic glutamate receptor group II pathway (P00040) | (i) B cell activation (P00010) | (i) EGF receptor signaling pathway (P00018) | (i) Apoptosis signaling pathway (P00006) | (i) General transcription regulation (P00023) | (i) Ubiquitin proteasome pathway (P00060) | (i) Alzheimer disease-amyloid secretase pathway (P00003) |
| (ii) GABA-B receptor II signaling (P05731) | (ii) T cell activation (P00053) | (ii) FGF signaling pathway (P00021) |  |  |  | (ii) Alzheimer disease-presenilin pathway (P00004) |
| (iii) Muscarinic acetylcholine receptor 2 and 4 signaling pathway (P00043) | (iii) Toll receptor signaling pathway (P00054) | (iii) TGF-beta signaling pathway (P00052) |  |  |  | (iii) Huntington disease (P00029) |
| (iv) Nicotinic acetylcholine receptor signaling pathway (P00044) | (iv) Inflammation mediated by chemokine and cytokine signaling pathway (P00031) | (iv) FAS signaling pathway (P00020) |  |  |  | (iv) Parkinson disease (P00049) |
| (v) 5HT1 type receptor mediated signaling pathway (P04373) | (v) Integrin signalling pathway (P00034) | (v) Heterotrimeric G-protein signaling pathway-Gq alpha and Go alpha mediated pathway (P00027) |  |  |  |  |
| (vi) Endogenous cannabinoid signaling (P05730) | (vi) Interferon-gamma signaling pathway (P00035) | (vi) Oxidative stress response (P00046) |  |  |  |  |
| (vii) Enkephalin release (P05913) |  | (vii) Ras Pathway (P04393) |  |  |  |  |
| (viii) Opioid prodynorphin pathway (P05916) |  |  |  |  |  |  |
| (ix) Opioid proopiomelanocortin pathway (P05917) |  |  |  |  |  |  |
| (x) CCKR signaling map (P06959) |  |  |  |  |  |  |
| (xi) Gonadotropin-releasing hormone receptor pathway (P06664) |  |  |  |  |  |  |
