# Supplementary figures and images for: Altered Expression of a Unique Set of Genes Reveals Complex Etiology of Schizophrenia
Source: Front Psychiatry. 2019 Dec 12;10:906. doi: 10.3389/fpsyt.2019.00906 (PMC6920214; doi:10.3389/fpsyt.2019.00906)

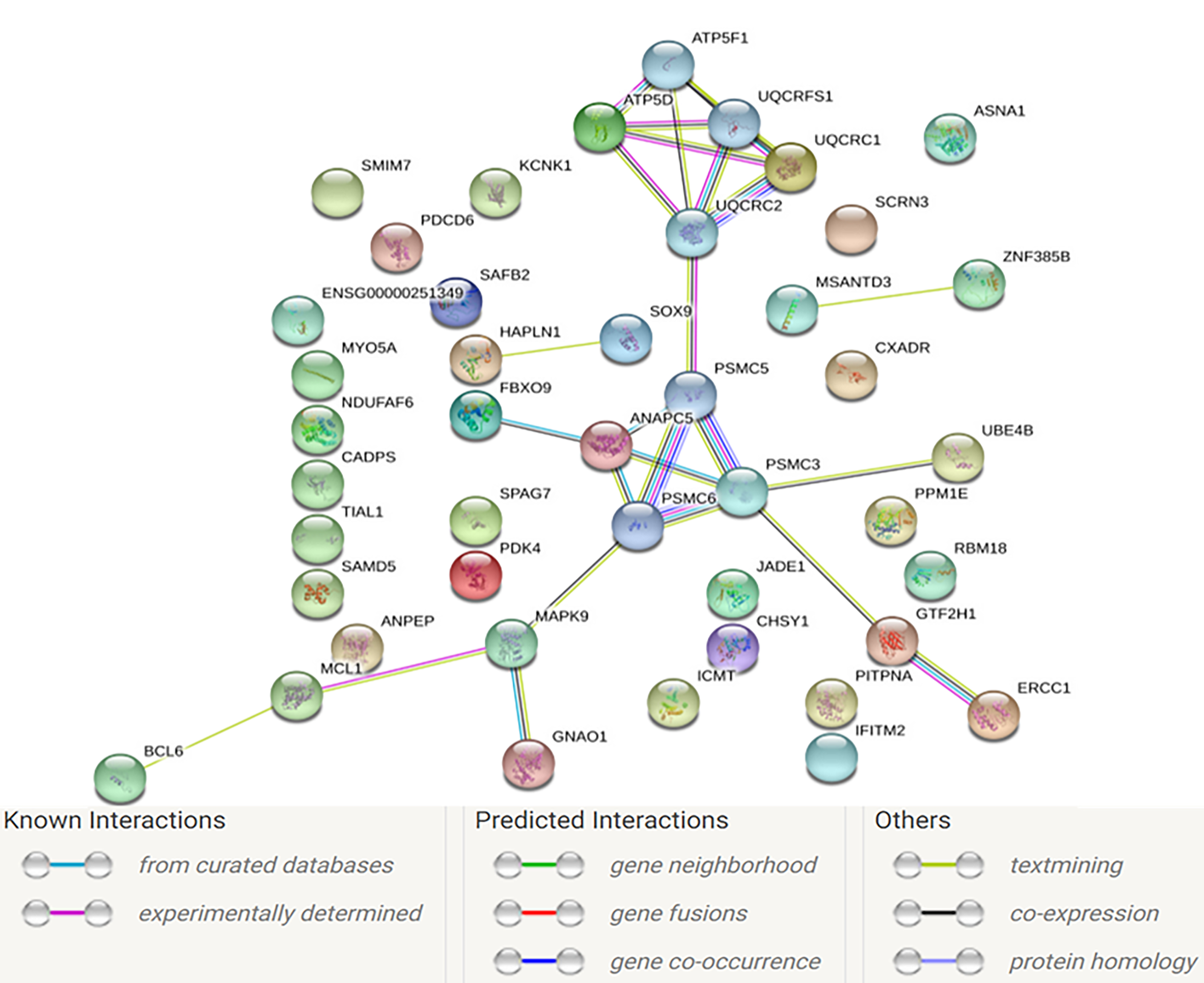

Supplement: Figure S1 — Protein Interactome Network Analysis of the gene set. Certain of the genes shown in the figure, such as JADE-1 and ENSG00000251349 or MSANTD3-TMEFF1, PSMC6, PSMC2, ATP5F1, are linker genes generated by the database and are not part of the original gene set. [file Image_1.tif]
